# A student’s perspective: utilizing the lived experience of healthcare leaders as a professional development tool

**DOI:** 10.3389/fmed.2025.1522732

**Published:** 2025-04-09

**Authors:** Michael A. Dewsnap, Sunitha E. Konatham

**Affiliations:** ^1^Department of Humanities in Medicine, Texas A&M University College of Medicine, College Station, TX, United States; ^2^Texas A&M College of Medicine, College Station, TX, United States

**Keywords:** professional development, leadership, lived experience, qualitative research, constructivist learning theory

## Abstract

Recent events like the COVID-19 pandemic or operational innovations such as the increased use of advanced providers have compelled physicians to take on additional roles like public health spokesperson or team leader. Lectures and workshops are common educational tools utilized to address these changing roles but require significant time, resources, and are often overshadowed by preference for personal experience. The purpose of this commentary is to suggest that the lived experience of healthcare leaders, as expressed through qualitative research-based interviews, offer an engaging educational tool for professional development of the interviewee and the interviewer, especially when a student is the interviewer. Through a student’s perspective, and building off a class project, three healthcare leaders were interviewed, and the responses analyzed. Common themes such as approachability, perspective taking, vision establishment, and team empowerment were identified. The student’s, and the supervising faculty co-author’s, reflections illustrate the impact of using this qualitative research approach to broaden their insights into the complexities associated with being a leader in a healthcare system that incorporates clinical care and educational requirements. However, scaling this tool to a medical school class, or student body, would require altering the method such as group interviews.

## Introduction

Physicians have increasingly found themselves in leadership roles beyond clinical care. For example, whether managing interprofessional healthcare teams or guiding the public through the COVID-19 pandemic, physicians are often called upon to lead in ways that extend far beyond their traditional medical training (1–6). Team leader and the more recent role due to the COVID-19 pandemic as a public health spokesperson are examples of roles physicians can take on but not ones their traditional training has necessarily addressed (7–9). In general, undergraduate medical education (UME) in the United States (US) has not effectively prepared physicians for such non-clinical leadership roles (5, 10–12).

Preparing medical students to lead in healthcare often occurs through formal curricular, or informal cocurricular, content in UME – if not on the job when these students enter practice. In a national study, Neeley et al. (13) found that only 34%, or approximately 48, of the 144 allopathic (i.e., MD) medical schools in the United States who responded to the survey had a formal leadership program, whether elective (33%) or required (35%) (13). More recently, Korndorffer et al. found that 62% (14) of 40 sampled US allopathic schools offered an optional professional development curriculum on leadership in medicine (11). Further evidence is needed, but it appears there has been little progress since Neeley et al.’s study (10, 13).

Arroliga and Stoller suggested that packed curricula, limited time, and competing priorities hinder progress by medical educators to provide professional development content like leadership education in medical schools (15, 16). Korndorffer’s et al.’s study confirmed time constraints for faculty and staff and added lack of buy-in as other barriers (11). Additionally, leadership training often relies on lectures, mentoring, or workshops requiring significant time or resources (11, 13) and limiting broad implementation. In short, due to evolving challenges in healthcare, there is a need to train medical students to address these challenges; however, current methods and tools are limited and often superseded by other pressing curricular demands (15, 16).

Could there be a more effective and economical alternative to traditional, didactic-heavy methods that would foster professional development around an area such as leadership? Framed by constructivist pedagogical framework, and through qualitative research-based interviews, the authors suggest that the lived experiences of healthcare leaders can serve as an educational tool to teach medical students about leadership in medicine and foster their overall professional development. After describing lived experience and constructivist learning theory, the methods for a course project are outlined. Lastly, an example from a medical student’s perspective of the educational impact of a course project on their, and the faculty co-author’s, leadership education and overall professional development is presented.

## Lived experience

Talking with front desk staff, taking a patient’s medical history, and discussing the plan of care with members of the healthcare team are a few of the common daily activities for physicians. These activities are referred to in qualitative research as *lived experience* or, “everyday life or social action” to explore phenomena like leadership in medicine [(17), pp. 26]. For example, an attending physician could be observed discussing a plan of care with a diverse healthcare team. While a simplistic scenario, the researcher might frame questions regarding *why* the attending physician chose specific communication strategies. In response, the attending physician may comment on the importance of accessible language and nonverbal cues. The physician may also discuss *how* positive and negative experiences shaped their own communication and leadership style. Everyday human interactions, such as the physician example above, illuminate the rich potential of the lived experiences. Qualitative research focuses on exploring *why* and *how* to derive meaning from their lived experiences rather than testing a hypothesis [(17), pp. 6].

## Constructivist pedagogical framework

The constructivist pedagogical framework, or learning theory, states that learners are not passive spectators but rather involved participants that bring prior knowledge, understanding, experiences, and preconceptions to learning (18, 19). For instance, a medical student engaging directly with a physician at a patient’s bedside, or one-on-one conversations with healthcare leaders offers a more engaging approach to learning than passively sitting in the back row of a lecture hall. These interactions transform learning from passive and transmissive with a student-observer focus to an active and constructive student-centered focus.

Before moving to a medical student’s example of the impact of this approach to learning about leadership in medicine, it is important to briefly state six principles of the constructivist learning theory noted below based on the recent work of Burns, Menchaca, and Dimock (20).

1. Learners bring prior knowledge, experience, and beliefs to learning.
2. Knowledge is constructed and informed by experiences and contexts.
3. Learning is a mental process that is active and reflective.
4. Learning is a developmental process.
5. Learning is a contextual and social process (21).
6. The learner is at the center of the process controlling and mediating information.

The philosophical roots in the constructivist inquiry paradigm and nuances of constructivist learning theory are complex but are reflected by the six principles stated above (19, 21, 27, 28). Through interviews and personable narrative style, the following sections detail the methods for a course project and illustrate learning from the lived experiences of three healthcare leaders for medical student, and co-author, Sunitha Konatham (SK).

## Course project methods

To explore leadership in medicine and specific to leading teams, SK interviewed three healthcare leaders as a project, and not an institutional study, in a required course during the first year of medical school. This project employed a convenience participant recruitment strategy. Participants invited to be interviewed as part of this project were based on accessibility and prior professional relationships with SK. Given the project’s exploratory nature, convenience recruitment allowed for efficient recruitment of individuals with direct experience in healthcare leadership roles while ensuring feasibility within the course’s time constraints. A Manager of Volunteer Services, a Cardiology Clinic Practice Administrator, and an Executive-Level Administrative Physician agreed to participate. Semi-structured interviews were conducted in-person or via ZOOM, each lasting 45–60 min. Interviews were either recorded with in-person detailed notes (i.e., Manager of Volunteer Services) or audio-recorded and transcribed verbatim. The interviews addressed four questions provided to all class participants by the co-author MD: (1) What does team leadership mean to you? (2) What are the team leader’s roles and responsibilities? (3) What are strengths and challenges of team-based care? and (4) What skills & strategies help enhance the performance of a team?

With the guidance MD through project instructions, SK conducted a thematic analysis by independently reviewing interview transcripts to identify patterns and key themes related to leadership in medicine. Using an inductive approach, themes emerged from the responses with not preset coding scheme. The analysis involved reading and re-reading transcripts, grouping significant statements into units and then into broader themes.

To enhance trustworthiness, the themes were triangulated across participants, ensuring consistency in redundant data to identify themes. Themes were shared with interviewees. As analysis was conducted only by SK, steps were taken to minimize bias through reflection conversations with MD and multiple transcript reviews.

## A medical student perspective on leadership from her interviews

Below are the leadership lessons SK learned from the lived experience of the healthcare leaders interviewed for the school project.

### Manager of Volunteer Services

“I cannot believe you used to shadow doctors and now you are studying to become one,” the Manager of Volunteer Services (MVS) remarked as she/he walked with SK from the lobby to the outside patio of the local hospital where SK volunteered as a high school student. Sitting in metal chairs across a shaded table, SK pulled out her notepad, eager to glean advice from the person who first showed her leadership in healthcare that inspired her passion for medicine.

SK and the MVS discussed SK’s time working at the front desk and visiting patients with a care cart to make their stays more comfortable, and SK shared how she initially saw these roles as less clinically important but grew to realize that friendly faces and willingness to serve were also a form of care. The MVS nodded, emphasizing that emotional intelligence is just as vital as cognitive intelligence when caring for those in pain.

The MVS also described her/his role as a “mediator” to facilitate collaboration between departmental leaders and volunteers. Department leaders seek more direct patient time, and volunteers want to contribute to a patient’s care, so they identify tasks volunteers can handle to free up providers to focus on patient care.

SK thanked the MVS for assigning her to the Medical Surgical Unit where she gained valuable insights into her desired career by observing how physicians interacted with patients and their families. SK commented, “I learned more about the type of doctor I wanted (or did not want) to be.”

The MVS chuckled and asked, “Do you want a tip?” She/he explained that this question sets a positive tone for advice or criticism. Support is vital with this strategy, as everyone should feel comfortable giving and receiving help. She/he demonstrates this support by keeping an open-door policy, developing connections with, and advocating for, volunteers.

As the lunch hour ended, SK asked the MVS to outline her/his job as a healthcare leader. She/he listed the five C’s guiding their leadership style, punctuating each one with a raised finger to count them off: compliance, compassion, calling, collaboration, and constructive criticism. SK and the MVS ended their time with a high-five, symbolizing how she/he had passed on those five attributes.

### Cardiology Clinic Practice Administrator

SK’s next meeting was with the Cardiology Clinic Practice Administrator’s (CCPA) on the same hospital’s second floor, where SK had shadowed during her undergraduate studies. The CCPA’s managerial experiences and formal education in business administration made her/him an ideal resource for learning more about leadership in medicine.

SK noted how the sunlight streaming in through the large windows contrasted with their old windowless office. The CCPA smiled and agreed, sharing that the decision to move offices was tougher than anticipated. While the new office was pleasant, she/he was no longer in the middle of the action and needed to intentionally, “go walk around and be involved in the day-to-day things that impact patient experiences” to build trust.

As a leader in the cardiology clinic, the CCPA set staff objectives without micromanaging, believing that true leadership inspires people to do the right thing without being asked. She/he also explained that allowing people to fail can be a learning opportunity, and leader can empower people to use mistakes positively.

The CCPA also shared an example of how she/he resolved an operational concern for the in-basket – an inbox for labs, imaging, and messages – allowing staff to work more effectively. Physicians expressed concerns about needing to reduce appointment times to meet growing demand, leaving less time for the in-basket. The team devised a triage system for the in-basket and the CCPA emphasized that everyone should, “practice at the top of their license.” For example, registered nurses (RNs) handle 60% of in-basket communications, while physician assistants (PAs) manage 30–35%, leaving 5–10% for the physician. She/he highlighted that listening is critical and does not always require having a solution. It is important to allow time and space for the team to voice concerns or develop a solution.

### Executive-Level Administrative Physician

The Executive-Level Administrative Physician (ELAP) is a pediatrician and preventive medicine specialist who earned a master’s in public health and over a decade of experience working in different public health positions at the county and state levels.

Between their busy schedules, SK and the ELAP settled on a 10:30 pm meeting over Zoom. While the ELAP searched the dimly lit hotel lobby for a Wi-Fi connection, SK perched her laptop on the counter in my medical school’s kitchen, eager for the ELAP’s authentic, and unfiltered, perspective on leadership in medicine.

Personal and professional experiences taught the ELAP that leadership as a physician is two-fold: positional and influential. She/he stated that positional leadership is founded on technical understanding in an area, whereas influential leadership is founded on interpersonal skills. However, she/he emphasized that authority is responsibility earned, not a right. Although leadership can be positional, an impactful leader influences those they lead by modeling positive behavior, building relationships, and equipping others to excel in their roles.

Many physicians enter medicine focused solely on patient care, often lacking the essential skills to take on administrative roles due to limited training in medical school. The ELAP explained that “the M.D. behind your name gives you credibility – if I say something, people listen (adding in jest, ‘and sometimes they should not).” To build credibility and level the hierarchy, she/he prefers being addressed by first name. She/he further emphasized that the team’s purpose is to help the community, not personal agendas, and stress the importance of healthy debate behind closed doors while presenting a united front publicly.

## Student interviewer’s reflection about the interviewing process

Informally interviewing these healthcare leaders gave me a better grasp of the complexities of leadership in healthcare and revealed the value of learning from lived experiences. I used to view myself as a leader because of my title on a student leadership team, but these interviews also reminded me that leadership is about relationships. Leaders can be found at all levels and positions in healthcare, as demonstrated by these leaders. Afterward, I conducted a basic comparative analysis, identifying commonalities and extracting excerpts to illuminate the participants lived experiences. I grouped the commonalities under the themes of approachability, perspective-taking, vision establishment, and team empowerment (Table 1).

**Table 1:** Common themes of leadership from interviews.

	Approachability	Perspective taking	Vision establishment	Team empowerment
Management of Volunteer Services (MVS)	Open door policy	Individual meetings with volunteers and employees	Common vision and goals for all personnel	Delegate non-clinical tasks to other personnel
Cardiology Clinic Practice Administrator (CCPA)	Listening without judgement	Understanding roles to identify overlap or conflict	Break down hospital goals into objectives	Encourage autonomy
Executive-Level Administrative Physician (ELAP)	First name-basis connection	“Management by walking around”	Data analysis, resource assessment, and political engagement	Providing resources, autonomy, and vision

Just as our medical knowledge is developed in the classroom and nurtured in the clinic, leadership skills can be cultivated by observing experiences of individuals working in healthcare. These interviews were completed during my preclerkship coursework, and now as a current third-year medical student rotating through various clerkship experiences, I have continued to identify these themes in the leadership styles of faculty. One key takeaway from these interviews is that leadership may seem like a soft skill, but it’s hard. Leadership is often regarded as a personality trait that is not easily measured or defined. Yet, students are expected to graduate with the ability to lead their peers and patients. Even those appearing naturally inclined for leadership need education, examples, and insights from the lived experiences of other healthcare leaders.

## Discussion

As SK established through her interview narratives, learning from the lived experiences of healthcare leaders broadened her understanding of leadership in medicine. The literature in medical education points to the broad use of qualitative research-based interviews to research phenomena such as student perspectives on outpatient education, the patient experience, or improving clinical outcomes (22–24). However, despite scant evidence in the nursing literature (14), utilizing lived experiences of healthcare leaders as an educational tool is largely absent.

Not only did SK hear what was stated in the interviews, SK synthesized the information and drew connections through themes communicated from each leader – supporting the efficacy of lived experiences as an educational tool. For instance, in-basket communication became an opportunity to deconstruct leader’s approach and provide a rich learning experience. Rather than learn about leadership in a lecture hall, SK engaged in meaningful discussion with the healthcare leaders, fostering mentoring relationships that proved beneficial to her professional development.

While the benefits of a constructivist-based approach to learning include active involvement of the learner, there are limitations. SK’s learning was based on only three healthcare leaders. While the small participant pool may limit generalizability, the goal was not broad representation but rather in-depth exploration of lived experiences. Next, this approach requires time from healthcare leaders to be interviewed; which may be difficult to secure. Lastly, SK’s existing relationships with the participants likely fostered mentoring discussions. Mentoring is widely regarded as another important method for training medical students (25) which was an advantage for SK but may not be for future students who did not have existing relationships with healthcare leaders.

Interviewing healthcare leaders can be educationally fruitful. However, scaling interviewing healthcare leaders to entire student body of 600+ students would require adjusting the methods Implementing lived experience interviews on a larger scale would require adjusting the methods. For example, instead of one-on-one interviews, a group of students in a course focused on the practice of medicine could interview a set group of healthcare leaders in person or virtually. Additionally, medical schools are increasingly implementing faculty-led longitudinal small groups, also known commonly as learning communities, for the entire student body to address aspects like mentoring, education, and wellbeing (26). For schools with these learning communities, another option is to use these groups as the setting for students to interview their faculty leaders (i.e., group-on-one). Learning communities could even trade leaders to give students the option to hear from multiple faculty. In groups, the students then could collaboratively construct an understanding of the interviews as well as commonalities, differences, strengths, and challenges between the lived experiences of the faculty interviewed – promoting critical thinking and further supporting the constructivist pedagogical framework.

## Conclusion

Traditional efforts to promote professional development (e.g., teaching leadership in medicine) often rely on passive methods such as seminars or workshops (11, 13). Moreover, medical students often elevate personal experience over the classroom teaching of leadership; opting to learn while “on the job.” Exploring the lived experiences of healthcare leaders through qualitative research-based interviews offers an engaging way for students to learn directly from those working daily in the medical field. In addition, the potential ancillary opportunities of mentoring and collaboration between faculty and students, as exemplified by SK’s example, offers additional educational benefits to enrich student learning.

With Artificial Intelligence (AI) becoming more integrated into medical practice, physicians will be tested again as to what role they will play in this next era of innovation. Medical educators must adapt not only in teaching the basic sciences (e.g., Anatomy), but also effectively prepare physicians for another change in the medical field. Actively learning at the bedside, one-on-one, or group-on-one conversations with healthcare leaders situates students at the center of a more engaged and practical learning process than at the back of a lecture hall.

## Data Availability

The original contributions presented in the study are included in the article, further inquiries can be directed to the corresponding author.

## References

[1] Centers for Disease Control [CDC]. (2023). CDC museum COVID-19 timeline. *David J. Sencer CDC Museum: In Association with the Smithsonian Institution*. Available online at:https://www.cdc.gov/museum/timeline/covid19.html.

[2] National Foundation for Infectious Diseases [NFID]. (2024). COVID-19. *National Foundation for Infectious Diseases*. Available online at:https://www.nfid.org/infectious-diseases/covid-19/.

[3] Perlis RH Ognyanova K Uslu A Trujillo KL Santillana M Druckman JN. Trust in physicians and hospitals during the COVID-19 pandemic in a 50-state survey of US adults. JAMA Netw Open. (2024) 7:e2424984–4. doi: 10.1001/jamanetworkopen.2024.24984, PMID: 39083270 PMC11292455

[4] Rotenstein LS Huckman RS Cassel CK. Making doctors effective managers and leaders: a matter of health and well-being. Acad Med. (2021) 96:652–4. doi: 10.1097/ACM.0000000000003887, PMID: 33332911

[5] Dickerman J Sánchez J Portela-Martinez M Roldan E. Leadership and academic medicine: preparing medical students and residents to be effective leaders for the 21st century. MedEdPORTAL. (2018) 14:1–8. doi: 10.15766/mep_2374-8265.10677, PMID: 30800877 PMC6342431

[6] Hofer AN Abraham JM Moscovice I. Expansion of coverage under the patient protection and affordable care act and primary care utilization. Milbank Q. (2011) 89:69–89. doi: 10.1111/j.1468-0009.2011.00620.x, PMID: 21418313 PMC3160595

[7] Morrison G Goldfarb S Lanken PN. Team training of medical students in the 21st century: would Flexner approve? Acad Med. (2010) 85:254–9. doi: 10.1097/ACM.0b013e3181c8845e, PMID: 20107351

[8] Snell AJ Briscoe D Dickson G. From the inside out: the engagement of physicians as leaders in health care settings. Qual Health Res. (2011) 21:952–67. doi: 10.1177/1049732311399780, PMID: 21343436

[9] Bohmer RMJ. Managing the new primary care: the new skills that will be needed. Health Affairs. (2010) 29:5. doi: 10.1377/hlthaff.2010.0197 20439899

[10] Matsas B Goralnick E Bass M Barnett E Nagle B Sullivan EE. Leadership development in US undergraduate medical education: a scoping review of curricular content and competency frameworks. Acad Med. (2022) 97:899–908. doi: 10.1097/ACM.0000000000004632, PMID: 35171123

[11] Korndorffer M Dewsnap MA Barry ES Grunberg N Musick DW Quinn JF. Pilot study exploring the presence of leadership curricula in undergraduate medical education. BMJ Lead. (2024) 8:340–7. doi: 10.1136/leader-2023-000957 38565276

[12] Theobald M. STFM prepares family medicine educators to lead. Ann Fam Med. (2015) 13:89–90. doi: 10.1370/afm.1749, PMID: 25583901 PMC4291274

[13] Neeley SM Clyne B Resnick-Ault D. The state of leadership education in US medical schools: results of a national survey. Med Educ Online. (2017) 22:1301697. doi: 10.1080/10872981.2017.1301697, PMID: 28298155 PMC5419299

[14] Bond C. A qualitative study: Exploring perceptions of leadership among nurses Franklin University (2021).

[15] Arroliga AC Huber C Myers JD Dieckert JP Wesson D. Leadership in health care for the 21st century: challenges and opportunities. Am J Med. (2014) 127:246–9. doi: 10.1016/j.amjmed.2013.11.004, PMID: 24280177

[16] Stoller JK. Commentary: recommendations and remaining questions for health care leadership training programs. Acad Med. (2013) 88:12–5. doi: 10.1097/ACM.0b013e318276bff1, PMID: 23267224

[17] Merriam SB Tisdell EJ. Qualitative research: A guide to design and implementation. Newark, NJ: Wiley (2015).

[18] Bada SO Olusegun S. Constructivism learning theory: a paradigm for teaching and learning. J Res Method Educ. (2015) 5:66–70. doi: 10.9790/7388-05616670

[19] Dennick R. Constructivism: reflections on twenty five years teaching the constructivist approach in medical education. Int J Med Educ. (2016) 7:200–5. doi: 10.5116/ijme.5763.de11, PMID: 27344115 PMC4939219

[20] Burns M Menchaca M Dimock V. Applying technology to restructuring and learning In: Computer support for collaborative learning. Gerry Stahl ed. (New York, NY: Routledge) (2023). 281–9. Available at: https://www.taylorfrancis.com/books/edit/10.4324/9781315045467/computer-support-collaborative-learning-gerry-stahl?refId=a5bae38a-1f6f-44a8-b894-215d51725e74&context=ubx

[21] University of Buffalo [UB]. (2024). Constructivism. *Office of curriculum, assessment, and teaching transformation*. Available at: Available online at: https://www.buffalo.edu/catt/teach/develop/theory/constructivism.html

[22] Barzegar M Faghihi SA Amini M Zarifsanaiey N Boushehri E. Outpatient education, a momentous in clinical education: a qualitative study of medical students’, faculty members’, and residents’ perspectives. BMC Med Educ. (2023) 23:719. doi: 10.1186/s12909-023-04694-3, PMID: 37789306 PMC10548757

[23] Tokumasu K Va P Obara H Rucker L. What medical students and residents learned from reflection through patients’ perspectives: a qualitative study. Int J Med Educ. (2024) 15:150–8. doi: 10.5116/ijme.6741.f16c 39641348 PMC11687375

[24] Steinbach TC Jennerich AL Çoruh B. Effective behaviors of leaders during clinical emergencies: a qualitative study of followers’ perspectives. Chest. (2024) 166:1141–50. doi: 10.1016/j.chest.2024.05.011, PMID: 38838955

[25] Wu J Olagunju AT. Mentorship in medical education: reflections on the importance of both unofficial and official mentorship programs. BMC Med Educ. London, UK: Springer Nature (2024) 24:1233. doi: 10.1186/s12909-024-06248-7, PMID: 39472896 PMC11523898

[26] Shochet R Fleming A Wagner J Colbert-Getz J Bhutiani M Moynahan K. Defining learning communities in undergraduate medical education: a national study. J Med Educat Curri Develop. (2019) 6:2382120519827911. doi: 10.1177/2382120519827911, PMID: 30937385 PMC6434432

[27] Moustakas C. Phenomenological research methods. Thousand Oaks, CA: Sage (1994).

[28] Rillo AG Martínez-Carrillo BE Castillo-Cardiel JA Rementería-Salinas JM. Constructivism: an interpretation from medical education. IOSR J Res Method Educ. (2020) 10:13. doi: 10.9790/7388-1003070112

